# Prolonged postoperative ileus in a patient with primary pneumatosis cystoides intestinalis: a case report

**DOI:** 10.1186/s40792-018-0431-6

**Published:** 2018-03-14

**Authors:** Hiroshi Tamura, Tatsuo Kanda, Tadasu Chida, Hitoshi Kameyama, Ukihide Tateishi, Toshifumi Wakai, Makoto Naito

**Affiliations:** 1 Department of Surgery, Sanjo General Hospital, Tsukanome, Sanjo 955-0055 Japan; 2 Division of Digestive and General Surgery, Niigata University Graduate School of Medical and Dental Sciences, Asahimachi-dori, Chuo-ku, Niigata, 951-8510 Japan; 3 Department of Radiology, Tokyo Medical and Dental University, Yushima, Bunkyo-ku, Tokyo, 113-8510 Japan; 4 Department of Pathology, Niigata Medical Center, Kobari, Nishi-ku, Niigata, 950-2022 Japan

**Keywords:** Paralytic ileus, Pneumatosis cystoides intestinalis, Hyperbaric oxygen therapy, Duodenum

## Abstract

**Background:**

Pneumatosis cystoides intestinalis (PCI) is a rare disease characterized by multiple gas-filled cysts in the intestinal wall and is associated with various comorbidities. We report herein a case of intractable paralytic ileus caused by primary PCI.

**Case presentation:**

A 73-year-old man visited out hospital complaining of abdominal pain and vomiting. He had been hospitalized twice for intestinal obstruction in the past 2 months. Based on his history of appendectomy, mechanical bowel obstruction caused by adhesion was diagnosed, and the patient underwent surgery. However, laparotomy revealed small bowel dilatation despite the absence of obstruction or stenosis. Multiple nodules were found in the wall of the dilated bowel loops. The dilated jejunum was excised. Histological examination revealed that the nodules were small gas-filled cysts, suggesting PCI. We made a diagnosis of ileus with underlying PCI and managed the patient conservatively. A large amount of nasogastric tube drainage continued for a long period postoperatively. The patient underwent relaparotomy 35 days after the first operation. The upper jejunum was markedly dilated, although no mechanical stenosis was found. The atonic, dilated jejunum was excised and the ileal stump was anastomosed to the duodenum in a double tract fashion. The patient underwent hyperbaric oxygen therapy because the ileus persisted postoperatively. His condition gradually improved and he was discharged 53 days after the second operation.

**Conclusions:**

Non-operative treatment is recommended for primary PCI of unknown etiology. Surgeons should be mindful of the possibility of primary PCI when considering surgical intervention for patients with bowel obstruction.

## Background

Pneumatosis cystoides intestinalis (PCI) is a rare disease characterized by the presence of multilocular gas-filled cysts in the intestinal wall. The etiology of PCI is still unclear, although many theories have been proposed. Secondary PCI, which is associated with bowel obstruction or necrosis, is an indication for urgent surgery, while surgical intervention is usually unnecessary for primary or idiopathic PCI. Despite a considerable number of case reports, primary PCI is still not widely recognized as a disease entity. We report here a case of primary PCI where the patient underwent surgery and had prolonged postoperative ileus.

## Case presentation

**Fig. 1:**
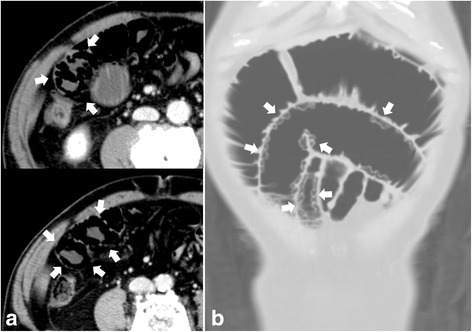
Computed tomography (CT) scan findings. Abdominal CT scans revealed dilatation of the small bowel from the duodenal third portion to the jejunum. Axial sections revealed dilated loops with a ring-like appearance due to intramural gas (**a**). Coronal section revealed multiple gas-filled cysts along the wall of the dilated jejunum (**b**). Arrows indicate pathognomonic intramural gas

A 73-year-old man with a history of appendectomy 50 years earlier visited our hospital complaining of abdominal pain and vomiting. The patient had been hospitalized twice in the past 2 months because of bowel obstruction. Abdominal X-ray showed dilated small-bowel loops with air-fluid levels. Computed tomography (CT) scans revealed dilation of the upper small bowel and the presence of intramural gas in a part of the dilated bowel segments. No causative lesion was identified (Fig. 1). We diagnosed small-bowel obstruction associated with intestinal pneumatosis secondary to increased intraluminal pressure. Surgery was indicated because the patient’s condition was serious enough to have warranted hospitalization three times in a period of only 2 months.

**Fig. 2:**
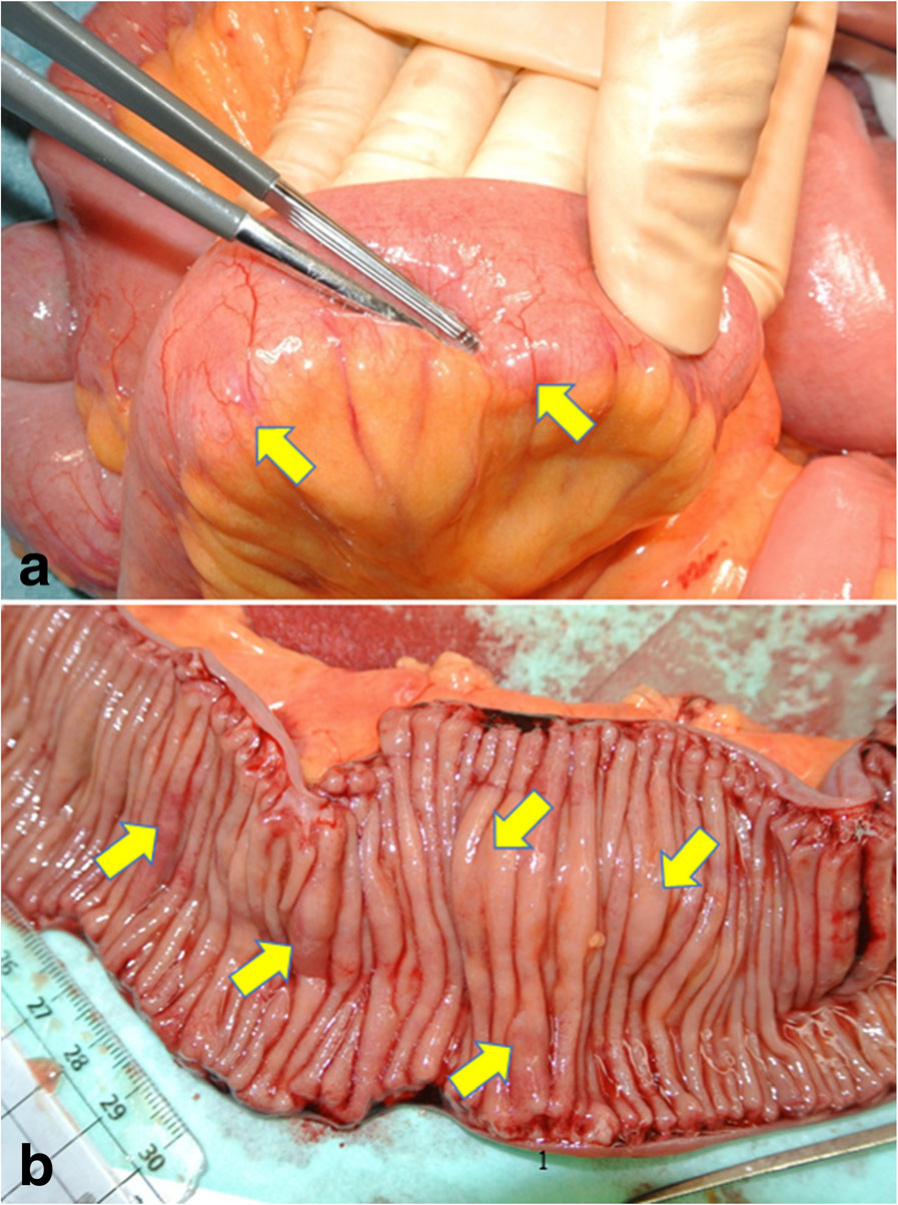
Surgical findings. The dilated bowel loops had multiple, small, intramural nodules. The gas-filled cysts were palpated as elastic nodules located in the bowel walls (**a**) and appeared like multiple submucosal tumors covered with normal mucosa in the resected specimen (**b**). Arrows indicate lesions

Laparotomy revealed that an approximately 160-cm loop of the proximal small bowel was dilated from the Treitz ligament. No significant stenosis was found. The dilated bowel had multiple small palpable nodules in the wall with areas of focal emphysema. Because confirmative diagnosis could not be made intraoperatively, we decided to partially resect the dilated small bowel, excising approximately 65 cm of the jejunum where the nodules were mainly distributed (Fig. 2).

**Fig. 3:**
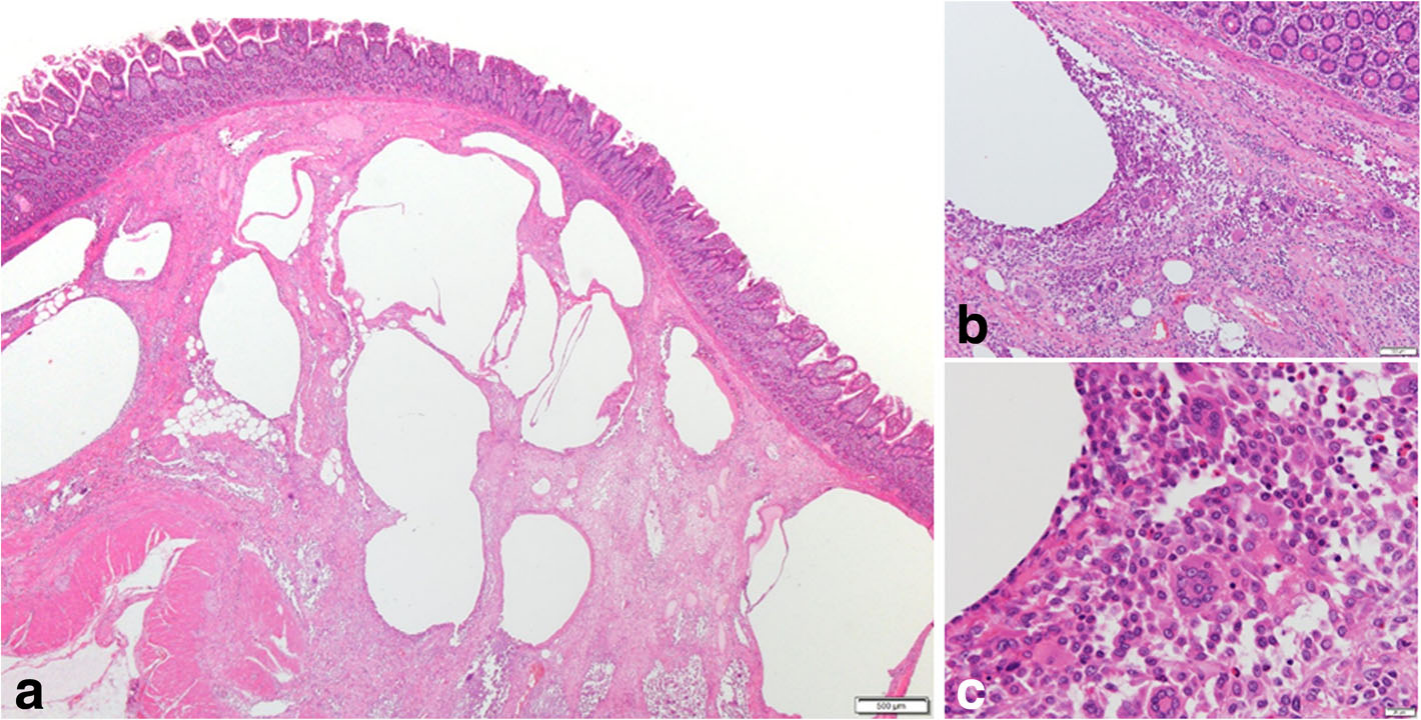
Microscopic findings. **a** Histopathological examination revealed the nodules to be multiple gas-filled cysts in the submucosa. **b**, **c** Non-specific inflammatory cells including macrophages and multinucleated giant cells infiltrated in the stroma (**b**, low-power view; **c**, high-power view)

Histopathological examination of the excised bowel revealed that the nodules were composed of multiple, gas-filled intramural cysts. Inflammatory cell infiltrates were noted in the stroma, although no specific finding was found in the cell population (Fig. 3).

**Fig. 4:**
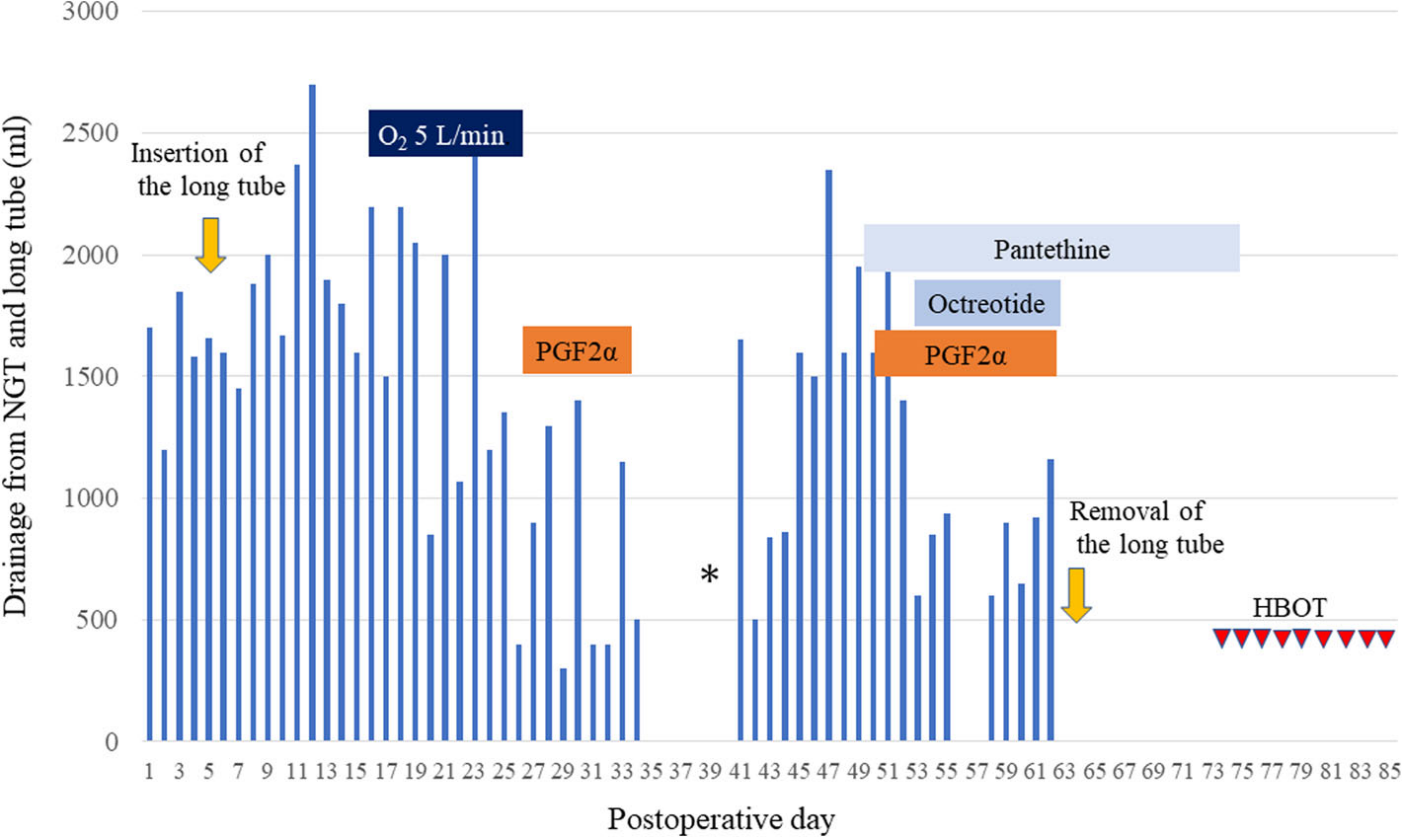
Long tube drainage and treatment course. High output discharge from a nasogastric tube (NGT) or a long tube continued despite oxygen insufflation. The days with no data on the amount of drainage are days when the long tube was temporarily clamped or removed. *HBOT* hyperbaric oxygen therapy, *PGF2α* prostaglandin F2α. An asterisk shows the day of the second laparotomy and triangles indicate the days when the patient underwent HBOT

**Fig. 5:**
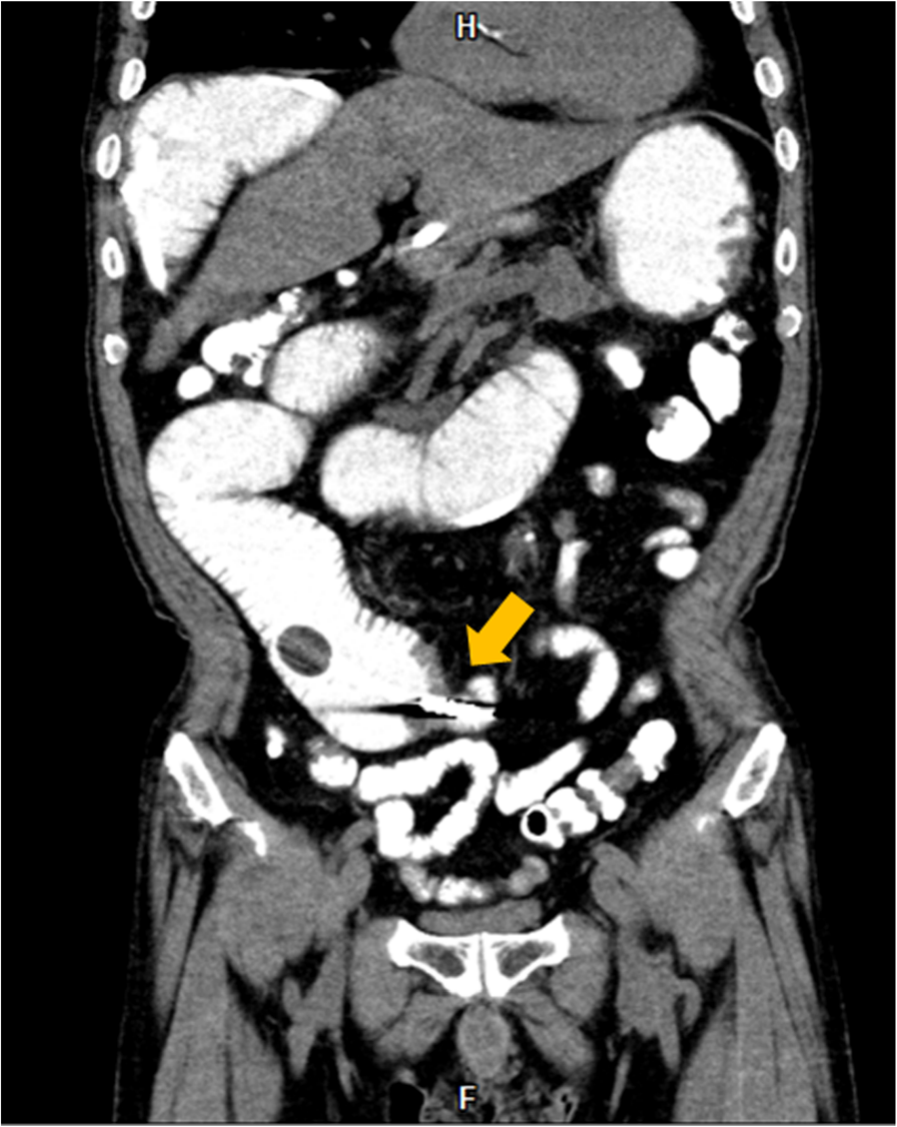
Computed tomography (CT) enterography. CT enterography suggested anastomotic stenosis, although water-soluble contrast medium passed through to the distal bowels. Arrow indicates caliber change at the jejunoileal anastomosis

High output discharge from a nasogastric tube (NGT), ranging from 0.9 to 2.7 L/day, was observed after the operation. The patient was diagnosed with postoperative ileus with underlying PCI and managed conservatively. The amount of drainage, however, did not decrease despite long-tube decompression and oxygen insufflation (Fig. 4). The patient was transferred to a university hospital for hyperbaric oxygen therapy (HBOT). However, CT enterography at the university hospital suggested anastomotic stenosis because the balloon of the long tube was retained there, although water-soluble contrast medium could pass (Fig. 5). Reoperation was recommended and the patient underwent laparotomy 35 days after the first operation.

**Fig. 6:**
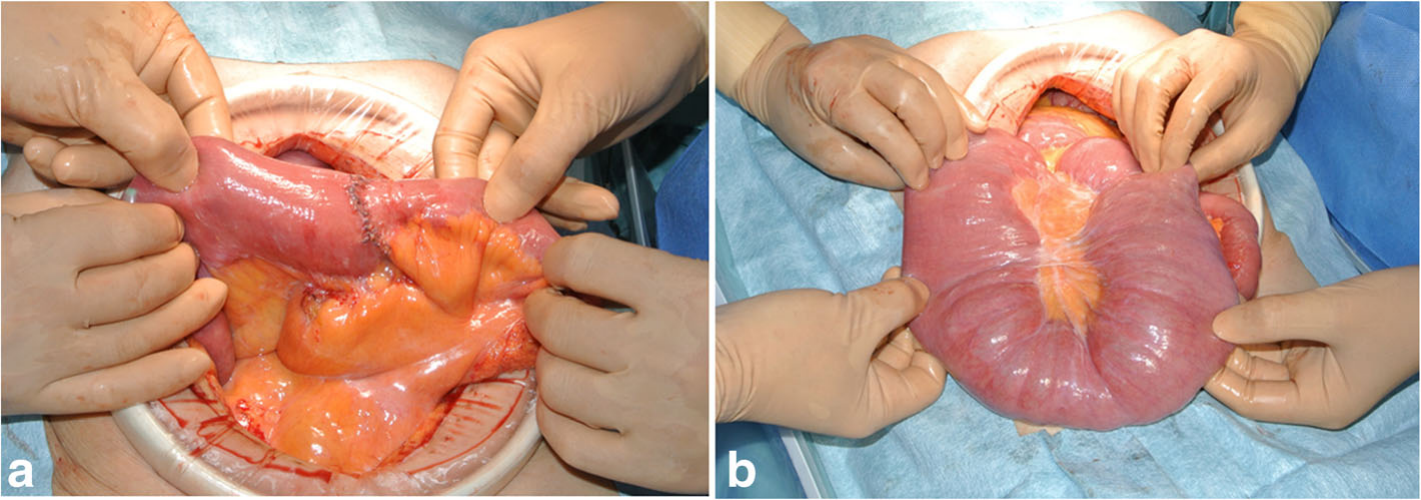
Intraoperative findings during the second operation. **a** The jejunoileal anastomosis was not stenotic. **b** The proximal small bowel was markedly dilated

On laparotomy, the proximal small bowel was noted to be markedly dilated although the jejunoileal anastomosis was not stenotic (Fig. 6). We divided the jejunum 15 cm distal from the Treitz ligament and excised the atonic, dilated jejunum, 36 cm in length. The proximal end of the ileum was anastomosed to the duodenal second portion in a double tract fashion, which bypassed the dilated third portion of the duodenum and the jejunal cuff. Histopathological examination revealed that the excised jejunum also had small gas-filled cysts, while the myenteric nerve plexuses were normally distributed.

The patient had prolonged ileus even after the second operation. Prostaglandin F2 alpha and long-acting octreotide were somewhat effective, but the effects were temporary. Endoscopic examination revealed that the passage created by duodenoileostomy was widely open and the fiberscope easily entered the ileal limb. The patient underwent HBOT for 9 days. Thereafter, the incidence of vomiting, which repeatedly occurred, gradually decreased. The patient resumed oral intake and was discharged 53 days after the second operation. Although a cyst-like dilatation of the third portion of the duodenum was seen on a follow-up CT scan conducted 15 months later, the patient’s quality of life is presently good, and he is able to tolerate a normal daily diet.

## Discussion

PCI is a disease that forms gas-filled cysts in the submucosa and subserosa of the intestinal tract [1]. The disease was first reported by Du Vernoi et al. in 1730 [2]. The etiology, however, remains unknown. Four major hypotheses have currently been proposed: (1) the mechanical theory, proposing that gas enters the digestive tract walls through mucosal injury in association with increased luminal pressure [3]; (2) the bacterial theory, proposing that gas-producing bacteria may cause intramural gas-filled cysts [4]; (3) the chemical theory, proposing that chronic exposure to chemicals, such as trichloroethylene, alpha-glucosidase inhibitors, or steroids, may impair the integrity of the mucosa [5]; and (4) the lung theory, proposing that the intramural bowel gas may originate from pneumomediastinum caused by alveolar rupture in cases of chronic obstructive pulmonary disease (COPD) [6].

The final diagnosis in the present case was primary PCI because surgery showed no mechanical obstruction and the patient had no history of any condition associated with PCI. The patient had previously been diagnosed as having COPD based on honeycomb-like changes seen on pulmonary CT scans. However, the fibrotic changes were limited to the pulmonary bases and the patient had no pulmonary symptoms. Asymptomatic COPD seemed unlikely to have a significant association with PCI.

The patient in this case had prolonged ileus. We believe that the initial laparotomy adversely affected the clinical course. In general, non-surgical treatment including HBOT is preferred for management of primary PCI. Treatment decision-making is not clear cut, however, because PCI sometime leads to life-threatening abdominal emergencies requiring urgent surgery. Treyaud et al. [7] retrospectively analyzed 149 patients in whom PCI was evident on multidetector CT and found the most frequent cause to be intestinal ischemia (53.7%), followed by infection (12.1%) and bowel obstruction (8.1%). Non-obstructive bowel dilatation was seen in only 6.7% of the patients. The overall mortality in that study was 41.6% and the presence of port-mesenteric venous gas (PVG) and decreased mural contrast enhancement were significant diagnostic radiological findings associated with bowel ischemia. Meanwhile, Morris et al. [8] analyzed patients with PCI at their institution between 2000 and 2007, and reported that non-operative management was selected for 50 patients (52%), of whom only 3 died. The authors found that a high APACHE II score was a significant risk factor for mortality in their study. Wayne et al. [9] classified 88 patients who showed PCI and/or PVG into three subgroups: acute mesenteric ischemia, mechanical causes, and benign idiopathic. Each of these subgroups accounted for about a third of the study cohort. The authors developed a scoring algorithm based on the data, taking into account a patient’s history, physical status, laboratory data, and radiological findings, and showed that the algorithm was useful in preventing non-therapeutic laparotomy. There is still no absolute indicator to suggest when urgent laparotomy is necessary for PCI patients. We have to decide whether surgery is indicated after careful consideration of the patient’s history, clinical status, and CT scan findings.

## Conclusions

Non-ischemic bowel diseases can also exhibit PCI. Medical treatment, including HBOT, is recommended for primary PCI, where the etiology is unknown. Surgeons should be mindful of the possibility of primary PCI when considering surgical intervention for patients presenting with bowel obstruction and PCI.

## Abbreviations

COPD: Chronic obstructive pulmonary disease
CT: Computed tomography
HBOT: Hyperbaric oxygen therapy
NGT: Nasogastric tube
PCI: Pneumatosis cystoides intestinalis
PGF2α: Prostaglandin F2 alpha
PVG: Port-mesenteric venous gas
